# Research on Single Nucleotide Polymorphisms Interaction Detection from Network Perspective

**DOI:** 10.1371/journal.pone.0119146

**Published:** 2015-03-12

**Authors:** Lingtao Su, Guixia Liu, Han Wang, Yuan Tian, Zhihui Zhou, Liang Han, Lun Yan

**Affiliations:** 1 College of Computer Science and Technology, Jilin University, Changchun, People’s Republic of China; 2 Key Laboratory of Symbolic Computation and Knowledge Engineering of Ministry of Education, Jilin University, Changchun, People’s Republic of China; 3 College of Computer Science and Information Technology, Northeast Normal University, Changchun, People’s Republic of China; The University of Chicago, UNITED STATES

## Abstract

Single Nucleotide Polymorphisms (SNPs) found in Genome-Wide Association Study (GWAS) mainly influence the susceptibility of complex diseases, but they still could not comprehensively explain the relationships between mutations and diseases. Interactions between SNPs are considered so important for deeply understanding of those relationships that several strategies have been proposed to explore such interactions. However, part of those methods perform poorly when marginal effects of disease loci are weak or absent, others may lack of considering high-order SNPs interactions, few methods have achieved the requirements in both performance and accuracy. Considering the above reasons, not only low-order, but also high-order SNP interactions as well as main-effect SNPs, should be taken into account in detection methods under an acceptable computational complexity. In this paper, a new pairwise (or low-order) interaction detection method IG (Interaction Gain) is introduced, in which disease models are not required and parallel computing is utilized. Furthermore, high-order SNP interactions were proposed to be detected by finding closely connected function modules of the network constructed from IG detection results. Tested by a wide range of simulated datasets and four WTCCC real datasets, the proposed methods accurately detected both low-order and high-order SNP interactions as well as disease-associated main-effect SNPS and it surpasses all competitors in performances. The research will advance complex diseases research by providing more reliable SNP interactions.

## Introduction

Detecting disease-associated SNPs is of great importance, because SNPs can affect diseases progression, how humans respond to drugs, vaccines, and other agents [1]. It is still a challenge to largely identify SNPs for any disease, one of the reasons is traditional single-loci test methods in GWAS can reveal only a few diseases causing SNPs, but more important reason is, SNPs do not work individually in complex diseases, rather, they cooperate with other SNPs to manifest a disease condition, which has been found in Osteoporosis [2] and breast cancer [3,4]. A recent report by Gerke et al. [5] also suggests that SNP interactions may carry more information about the phenotype than those observed from individual SNPs alone. Researchers are now focusing on detecting such SNP groups that are strongly associated with the disease phenotype. However, previous methods could not efficiently capture all those SNP interactions, especially when marginal effects of disease loci are weak or absent. Meanwhile, there are more than 10 million SNPs in the human genome, traditional two-locus based association studies are facing crucial computational burden, even worse of three or high-order-locus studies.

A number of methods have been proposed to detect pairwise interactions between SNPs, including Exhaustive algorithms (Nelson et al. [6]), Multifactor Dimensionality Reduction methods (e.g. MDR [7–10]), regression methods (e.g. Lasso Penalized Logistic Regression [11–13]), heuristic methods (Carlborg et al. [14], SNPHarvester [15]), mutual information method (e.g. SNPsyn [16]), as well as many other methods [6,17–20]. Exhaustive algorithms can find out all significant SNP interactions by enumerating out all possible SNP combinations, but have problems of long running time and poor scalability in dealing with large SNP datasets. The MDR method [7] is inspired by the combinatorial partitioning method, which can effectively reduce the genotype predictors from *n* dimensions to one dimension. However, its amount of calculation is excessive when dealing with more than 10 SNPs [21]. Regression methods have been pointed out as appropriate methods to estimate the strength of association between a predictor and disease [13]. However, they have limited performance in modeling high-order interactions [12]. To improve efficiency, two-stage approaches were used as a common heuristic strategy [15], that is, a subset of potential interacting SNPs is selected as candidate SNPs in the first stage, then analyzing interactions among them in the second stage. Despite the high speed efficiency such methods achieved, SNPs in low marginal effects are likely to be discarded in stage one. So, heuristic methods cannot guarantee to find the optimal solution. Other methods have low detection accuracy or performance when high-order SNP interactions are taken into consideration. In short, there are three major drawbacks of the methods mentioned above. Firstly, they were designed only for pairwise interactions detection, high-order interactions were not enough taken part into consideration according to their importance. Secondly, with the amount of data increasing, they dropped quickly in performance and accuracy. Thirdly, they all performed poorly especially when marginal effects of disease loci were weak or absent. SNPsyn [16] implemented an information theoretic approach for synergistic interaction analysis, which did not require users to specify which gene interaction models to test. It can use one or more GPUs to speed-up processing significantly. It can not only detect SNP interactions but also allow users to identify "main-effect" SNPs, *i*.*e*., SNPs that by themselves carry the most information of the related disease. However, SNPsyn does not take linkage disequilibrium between SNPs into consideration. There is an urgent need to develop new methods, with an acceptable computational complexity, for pairwise especially high-order SNP interactions detection. Furthermore, main-effect SNPs need to be measured from a new point of view.

In this study, we firstly propose a pairwise (or low-order) SNP interaction detection method IG (Interaction Gain), which is an extension of information gain in information theoretic [22,23]. Low-order SNP interactions will be parallel detected with IG from the data, by doing this, IG can scale to large datasets. Interaction gain expresses the amount of information bound up in a set of variables, beyond that which is presented in any subset of those variables. Contrary to other approaches, IG does not require users to specify which disease models to test in SNP interaction analysis, instead, it discovers them from data itself. Interaction gain of any pair of SNPs are calculated in parallel rather than only calculated heuristically. So, IG performs well even when marginal effects of disease loci are weak or absent. Instead of enumerating out all possible SNP combinations, high-order SNP interactions are efficiently detected by finding functional modules of SNP-SNP interaction network by using network clustering algorithms. In fact, SNP-SNP interaction network has similar topology structure as complex networks. By finding functional modules, we can achieve much higher speed while keeping high accuracy. With the SNP-SNP interaction network, finding main effective SNPs can be done by finding key nodes, which are measured by their degree and betweenness centrality. By using a number of simulated datasets and four WTCCC [24] real datasets, the performance of proposed methods were evaluated. Compared with previous methods, the proposed methods accurately detected out almost all forms of SNP interactions and surpassed all competitors in performances. The research will advance the complex diseases study by providing more reliable SNP interactions.

## Materials and Methods

### WTCCC datasets

**Fig 1:**
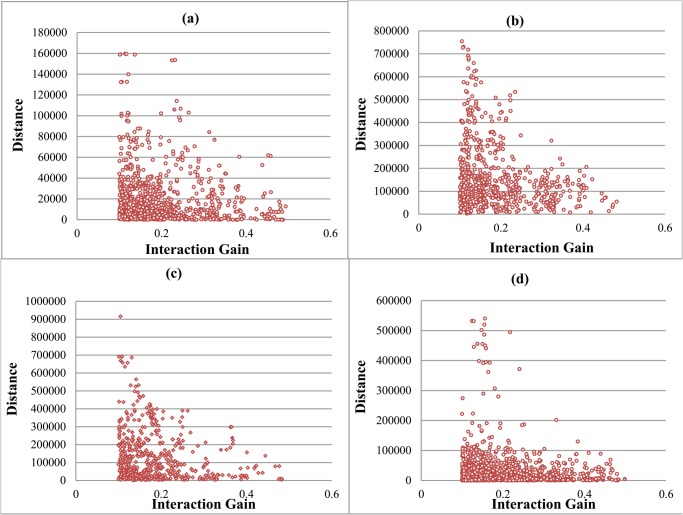
Relationship between interaction gain value and SNP base pair distance. (a) Interaction Gain value is calculated using SNPs all located in the area between genes. (b) Interaction Gain value is calculated using SNPs all located on the same gene. (c) Interaction Gain value is calculated using SNPs one locates on a gene and the other locates in the area between genes. (d) Interaction Gain value is calculated using SNPs all located on genes but on two different genes. We set *α* = 0.1 during all the computation.

SNP interactions are scanned on the publicly available dataset of Hypertension (The case individuals including 2000 patients in the British population, The control individuals came from two sources: 1,500 individuals from the 1958 British Birth Cohort (58C) and 1,500 individuals selected from blood donors recruited as part of this project (UK Blood Services (UKBS) controls). Both cases and controls are genotyped using the Affymetrix GeneChip 500k Mapping Array Set) from the Wellcome Trust Case Control Consortium (WTCCC) [24] which contains 511104 SNPs in all. Other WTCCC datasets, including bipolar disorder (BD), coronary artery disease (CAD), and type 2 diabetes (T2D), are also used in our experiments. Details of those datasets are presented in Table 1. Compared with SNPs from two different chromosomes, SNPs on the same chromosome tend to have much higher IG values. Besides, when SNPs are located on the same chromosome, if two SNPs have very high IG value then the distance between them is certainly very short (shown in Fig. 1). In other words, SNPs prone to interact with their physically close SNPs. However, when two SNPs are close to each other, a higher IG value may be obtained due to LD (linkage disequilibrium) existed between them. To deal with this problem, LD values between any SNP pairs of the SNP-SNP interaction network are calculated using SNAP (http://www.broadinstitute.org/mpg/snap/ldsearchpw.php) [25]. All the SNP pairs whose *r* ^2^ > 0.8 are discarded (Unless specified otherwise, we set r^2^ = 0.8, which works well in practice.). Considering the relationships between IG value and their base pair distance, four forms of IG values were calculated. As shown in Fig. 1, very high interaction gain values can be achieved only when two SNPs are physically close. So, only using SNPs located on the same chromosome to conduct our experiment will ensure accuracy of the final results.

**Table 1:** Details of real data sets got from WTCCC.

Data Sets	SNP	Case/Control	Chromosome
BD (bipolar disorder)	6865	1998/3000	21
HT (hypertension)	6207	2000/3000	22
CAD (coronary artery disease)	6114	1988/3000	19
T2D (type 2 diabetes)	4847	1999/3000	14

### Simulated datasets

Four kinds of epistatic models [20] are used to generate simulated datasets, their odds tables are given in Table 2. Model 1 is a multiplicative model. Model 2 is an epistatic model which has been used to describe handedness and the color of swine. Model 3 is a classical epistasis model. Model 4 is the well-known XOR model. In simulation, once the disease prevalence value *P*(*D*) and the genetic heritability value *h* ^2^ are specified, the parameters *δ* and *t* can be numerically solved (*δ* represents the impact value of the genotype at SNP location when there is no epistasis between SNPs; *t* represents the change of impact value when there are interactions between SNPs.). More specifically, we set *h* ^2^ = 0.03 for model 1 and *h* ^2^ = 0.02 for models 2, 3, 4. *P*(*D*)is set to 0.1 for all the models as in [15,20]. Genotype data is generated under the condition of the Hardy-Weinberg Equilibrium. The minor allele frequency (MAFs) of disease-associated SNPs is set to 0.1, 0.2, and 0.4. The MAFs of unassociated SNPs are generated uniformly between [0.05,0.5]. 100 datasets are simulated under each parameter setting of each disease model. Each dataset contains 1000 SNPs (x1 x2 … x1000, each has three values 0, 1, 2. The true interaction is between x1 and x1000). To take sample size into consideration, 1600 samples with 800 cases and 800 controls are simulated respectively.

**Table 2:** Four models used for simulated data generation.

Model 1	bb	Bb	BB	Model 2	bb	Bb	BB
aa	δ	δ	δ	aa	δ	δ (1+t)	δ (1+t)
Aa	δ	δ (1+t)	δ (1+t)^2^	Aa	δ (1+t)	δ	δ
AA	δ	δ (1+t)^2^	δ (1+t)^4^	AA	δ (1+t)	δ	δ
Model 3	bb	Bb	BB	Model 4	bb	Bb	BB
aa	δ	δ	δ (1+t)	aa	δ	δ (1+t)	δ
Aa	δ	δ (1+t)	δ	Aa	δ (1+t)	δ	δ (1+t)
AA	δ (1+t)	δ	δ	AA	δ	δ (1+t)	δ

*δ* represents the impact value of the genotype at SNP location when there is no epistasis between SNPs; *t* represents the change of impact value when there are interactions between SNPs.

### IG (Interaction Gain)

Interaction gain is defined as the difference between the actual decrease in entropy achieved by the joint attribute (X; Y) and the expected decrease in entropy with the assumption of independence between attributes X and Y. The higher the value, the more information is gained by joining the attributes together in comparison with the information gained from single attributes.

#### a. Definitions and notations

Matrix *X*_*N×M*_ represents the cohorts of *N* individuals (including both cases and controls) typed at *M* polymorphic sites, where each entry {0,1,2}in the matrix represents the number of minor alleles at a SNP site, rather than the presence or absence of a minor allele. *X*_*i*,*v*_ represents the genotype carried by individual *i* at variant site *v*. *X*_⋅*v*_ (0) = |{*i* | *X*_*i*,*v*_ = 0}|, *X*_⋅*v*_ (1) = |{*i* | *X*_*i*_,_*v*_ = 1}| and *X*_⋅*v*_ (2) = |{*i* | *X*_*i*_,_*v*_ = 2}| represent the number of individuals carrying genotype 0, 1, 2 respectively at the variant site *v*. Therefore, P_*v*_ (0) = *X*_⋅*v*_ (0) / *N* is the corresponding *0-frequency* of *v*, while *P*_*v*_ (1) and *P*_*v*_(2) denote the *1-frequency* and *2-frequency* of *v* respectively. Considering the case of two SNPs *v*_1_ and *v*_2_ with, for example, genotypes *AA* (0), *AC* (1), *CC* (2) and *TT* (0), *TG* (1), *GG* (2) respectively. Consequently, there are 9 genotype combinations between *v*_1_ and *v*_2_ defined as follows:

*X*_*i⋅v*_1_*v*_2__ = 00 if *X*_*i⋅v*_1__ = *AA* and *X*_*i⋅v*_2__ = *TT* of individual *i* at locus v_1_ and v_2_
*X*_*i⋅v*_1_*v*_2__ = 01 if *X*_*i⋅v*_1__ = *AA* and *X*_*i⋅v*_2__ = *TG* of individual *i* at locus v_1_ and v_2_
*X*_*i⋅v*_1_*v*_2__ = 02 if *X*_*i⋅v*_1__ = *AA* and *X*_*i⋅v*_2__ = *GG* of individual *i* at locus v_1_ and v_2_
*X*_*i⋅v*_1_*v*_2__ = 10 if *X*_*i⋅v*_1__ = *AC* and *X*_*i⋅v*_2__ = *TT* of individual *i* at locus v_1_ and v_2_
*X*_*i⋅v*_1_*v*_2__ = 11 if *X*_*i⋅v*_1__ = *AC* and *X*_*i⋅v*_2__ = *TG* of individual *i* at locus v_1_ and v_2_
*X*_*i⋅v*_1_*v*_2__ = 12 if *X*_*i⋅v*_1__ = *AC* and *X*_*i⋅v*_2__ = *GG* of individual *i* at locus v_1_ and v_2_
*X*_*i⋅v*_1_*v*_2__ = 20 if *X*_*i⋅v*_1__ = *CC* and *X*_*i⋅v*_2__ = *TT* of individual *i* at locus v_1_ and v_2_
*X*_*i⋅v*_1_*v*_2__ = 21 if *X*_*i⋅v*_1__ = *CC* and *X*_*i⋅v*_2__ = *TG* of individual *i* at locus v_1_ and v_2_
*X*_*i⋅v*_1_*v*_2__ = 22 if *X*_*i⋅v*_1__ = *CC* and *X*_*i⋅v*_2__ = *GG* of individual *i* at locus v_1_ and v_2_

Similarly as in single loci situation, *X*_⋅*v*_1_*v*_2__ (00) = |{*i* | *X*_*i⋅v*_1_*v*_2__ = 00}| represents the number of individuals that carry genotype 0 at *v*_1_ and genotype 0 at *v*_2_ (and analogously for *X*_⋅*v*_1_*v*_2__ (01),…,*X*_⋅*v*_1_*v*_2__ (22)). *P*_*v*_1_*v*_2__ (00) = *X*_⋅*v*_1_*v*_2__ (00) / *N* is the corresponding 00-frequency of *v*_1_ *v*_2_ (and *P*_⋅*v*_1_*v*_2__ (01),…, *P*_⋅*v*_1_*v*_2__ (22) are calculated correspondingly).

The most fundamental concept of information theory is the entropy. The entropy [26] of a random variable *X* is defined by Eqn. (1):

$$H(X)=-\sum_{x}p(x)\log p(x)$$ (1)

Where, *p(x)* is the marginal probability distribution function of variable *X*.

For two random variables *X* and *Y*, the joint entropy is defined by Eqn. (2):

$$H(X,Y)=-\sum_{x,y}p(x,y)\log p(x,y)$$ (2)

Where, *p(x*, *y)* is the joint probability distribution function of random variables *X* and *Y*.

The mutual information or information gain of variables *X* and *Y* is defined by Eqn. (3):

$$I(X;Y)=\sum_{x,y}p(x,y)\log\frac{p(x,y)}{p(x)p(y)}$$ (3)

The conditional mutual information of *X* and *Y* given *Z* is defined by Eqn. (4):

$$I(X;Y|Z)=\sum_{xyz}p(x,y,z)\log\frac{p(x,y|z)}{p(x|z)p(y|z)}$$ (4)

Where, *p*(*x*,*y* | *z*) is the conditional joint probability distribution function of variables *X*, *Y*, *Z*; *p*(*x* | *z*) and *p*(*y* | *z*)are conditional probability distribution functions of variables *X*, *Y* given *Z* respectively.

Using interaction gain as the metric to evaluate whether two SNPs are associated with the phenotype arises as a natural option. Two SNPs (X, Y) are associated with the phenotype P with a user-defined threshold α (Unless specified otherwise, we set α = 0.1, which works well in practice. The role of α is further discussed in parameter discussion section) if and only if I(X; Y; P)>α. I is the interaction gain and expresses the amount of information bound up in a set of variables, beyond that which is present in any subset of those variables, I(X; Y; P) = I(X; Y|P)-I(X; Y). Combined with Equation (3) and Equation (4), we can get Equation (5). If the information that SNP X provides about phenotype P is higher if we know SNP Y than it is if we do not know SNP Y, then this additional information is the interaction gain between the SNP X and Y with respect to phenotype P. The interaction gain of two SNPs i.e. *SNP*1, *SNP*2 with respect to phenotype *P* is defined in Eqn. (5):

$$I(SNP1;SNP2;P)=\sum_{x,y,c}p(x,y,c)\log\frac{p(x,y,c)p(c)}{p(x,c)p(y,c)}-\sum_{x,y}p(x,y)\log\frac{p(x,y)}{p(x)p(y)}$$ (5)

Where, *x* takes the values of 0, 1 or 2 (0 for homozygous dominant;1 for heterozygous; 2 for homozygous recessive); *y* also takes values from {0,1,2}; *c* takes values of 0,1 (0 for case; 1 for control).

#### b. Mapping SNPs on gene or between gene

The number of SNP combinations grows exponentially with the increase of the order of interaction (i.e. number of SNPs in combination) and the amount of SNPs in datasets. For example, given 6207 SNPs on chromosome 22, there are 19260321 pairwise SNP-SNP interactions, let alone higher-order interactions. Heuristic, non-exhaustive search requires shorter running time, but cannot guarantee to detect all significant interaction pairs. In this paper, we did not devise a new fast computing algorithm; rather, we calculated all interaction gain values in parallel to accelerate our computations. More specifically, SNPs locate on the same chromosome were firstly divided into two groups according to their chromosome bases location relationship with genes on that chromosome. As a result, of the 6207 SNPs of HT on chromosome 22, 2869 SNPs were mapped on 330 genes. Others located in the intergenic regions. The mapping results of other datasets are presented in Table 3.

**Table 3:** Mapping results of all the SNPs researched.

Data sets	Chromosome	All	On Gene	Between Gene	Gene
HT	22	6207	2869	3338	330
BD	21	6865	3089	3776	280
CAD	19	6114	3507	2607	1028
T2D	14	4847	1804	3043	192

All: the number of SNPs considered; On Gene: the number of SNPs mapped on genes; Between Gene: the number of SNPs mapped between genes; Gene: the number of genes mapped by SNPs

#### c. Workflow of IG

IG works in the following steps: Firstly, SNPs locate on the same chromosome are divided into two groups by mapping them on genes or between genes. Secondly, five forms of SNP-SNP interactions (SNPs within a certain gene, SNPs locate on two different genes, SNPs all locate between genes, SNPs locate on one gene with SNPs locate between genes, SNPs locate on one chromosome with SNPs on another.) are tested in parallel using Eqn. (5). Thirdly, LD values of all the SNP pairs are calculated using SNAP (http://www.broadinstitute.org/mpg/snap/ldsearchpw.php) [25]. All the SNP pairs whose r^2^ bigger than 0.8 are discarded. Finally, potential SNP-SNP interactions are selected according to threshold value α and are represented into a SNP-SNP interaction network. By executing those steps, IG can achieve high speed-up while maintaining high accuracy.

### SNP interaction detection

#### a. Network construction

**Fig 2:**
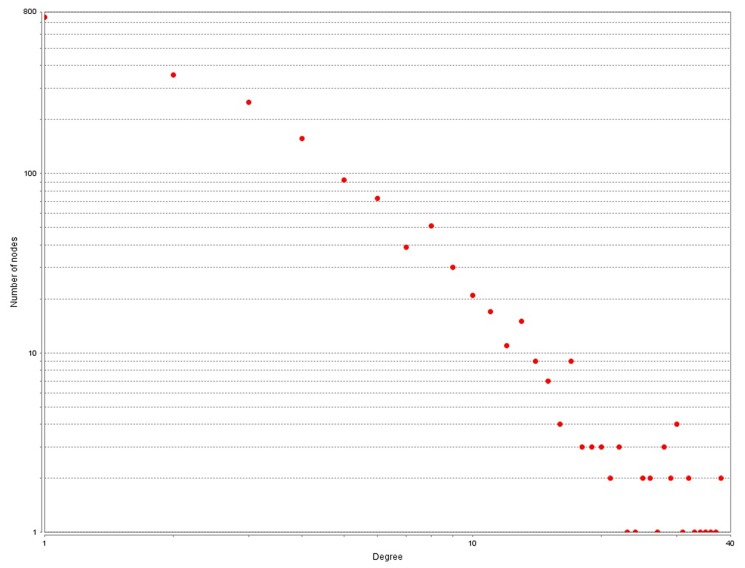
Node degree distribution of SNP-SNP interaction work constructed with *α* = 0.1. Degree is calculated by counting the edges of a SNP in the network.

**Fig 3:**
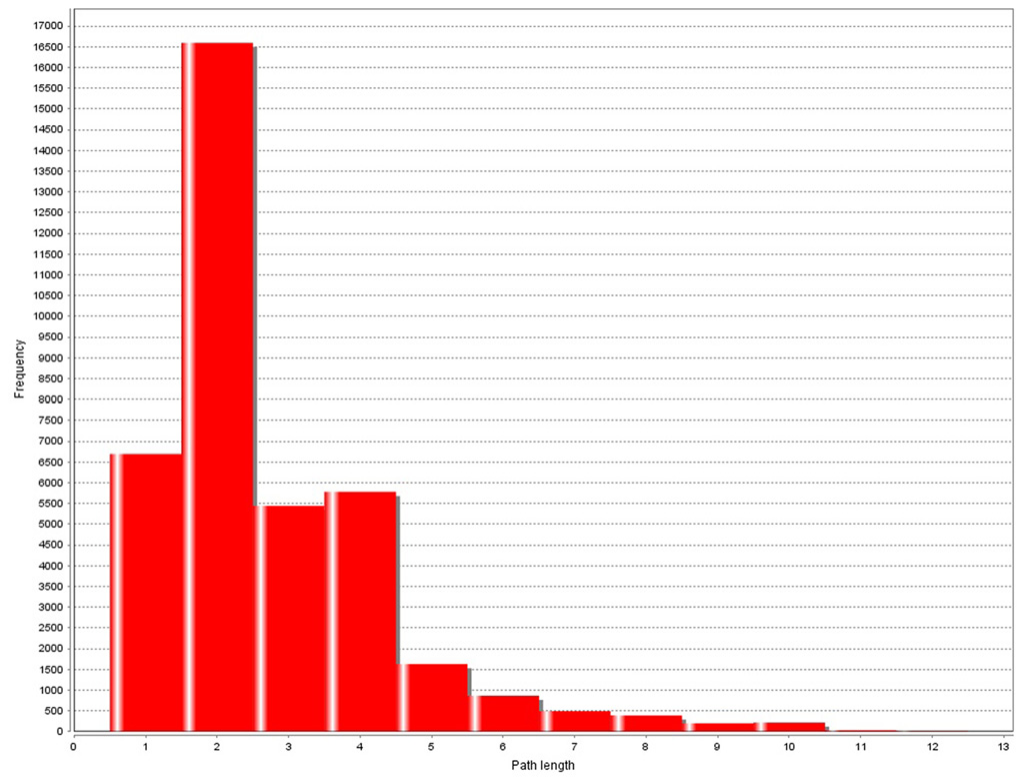
Shortest path length distribution of SNP-SNP interaction network constructed with *α* = 0.1. Shortest path means the shortest path between any two SNPs among all the possible paths. Frequency means how many SNP pairs have a certain shortest path length.

SNP pairs with interaction gain values bigger than α will be selected to construct the SNP-SNP interaction network, otherwise, they are discarded. As a result, 2676 and 2789 pairs of SNP-SNP interactions were selected respectively from the HT and T2D detection results. The degree and shortest path length distribution of the SNP-SNP interaction network are depicted in Fig. 2 and Fig. 3 respectively.

**Fig 4:**
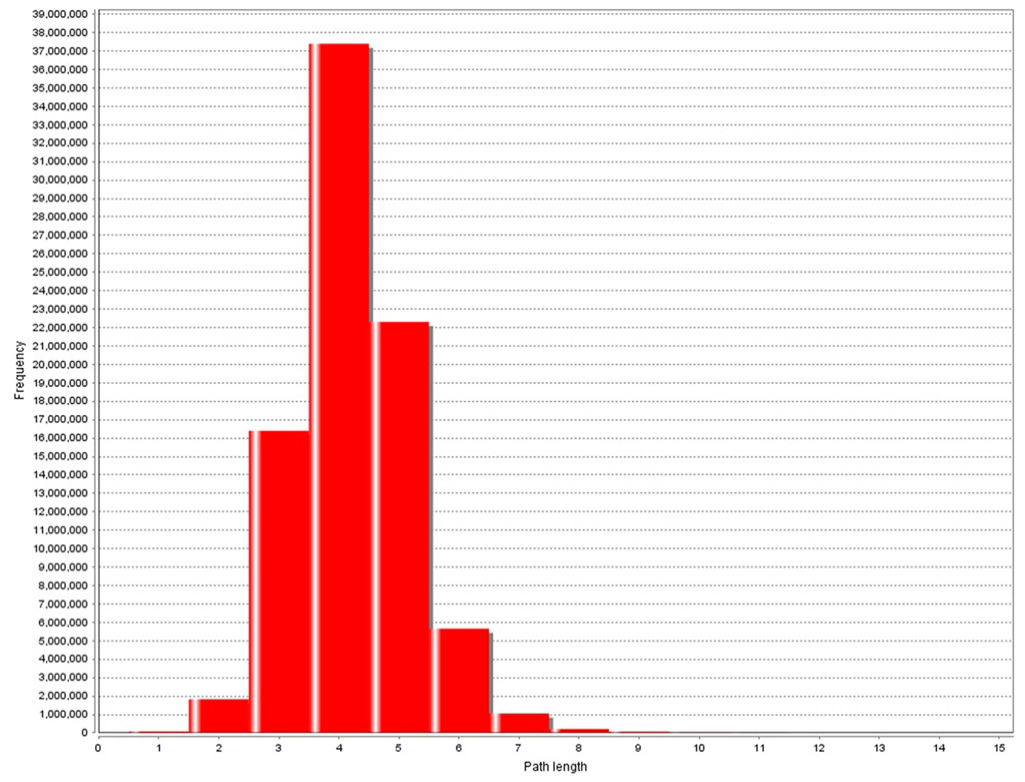
Shortest path length distribution of PPI network. Shortest path means the shortest path between any two proteins among all the possible paths. Frequency means how many protein pairs have a certain shortest path length.

As shown in Fig. 2, the node degree follows the power law distribution just as it in PPI networks. Of the 1649 SNPs in the network, only few of them have very high degrees. The average degree is about 3.4. However, the path length distribution has some differences compared with those in other complex networks. The path length distribution of SNP-SNP interaction network is asymmetric and the common length is 2, which is 4 in PPI networks (Fig. 4). In fact, pairwise interactions are most common in SNP-SNP interaction network, but at the same time few high order interactions also exist. Besides, in PPI networks path length usually shorter than 9, while in SNP-SNP interaction networks this value can reach to 11.

#### b. SNP functional module detection

High-order SNP interactions are detected by finding functional modules rather than by enumerating out all possible SNP combinations. According to previous analysis, SNP-SNP interaction network has some similar properties as complex networks [27]. One important characteristic of complex network is that it contains function modules (complexes) within the network. Predicting function modules from complex networks has long been studied and methods mainly include the following three categories: (1) Methods based on seed node expansion [28]. Such methods predict complexes in two stages, firstly, finding out seed nodes; secondly, expanding those seed nodes. Different strategies used in seed node selection and expansion affect performances of those methods significantly. (2) Methods based on hierarchical clustering [29,30]. Such methods represent the entire network in a tree like structure and find functional modules by cutting the whole tree at different levels. (3) Heuristic methods [31]. This kind of methods usually have better performance due to introducing additional information about nodes besides the topology of complex networks. Since SNPs tend to interact with SNPs who are close to them. So, methods based on seed node expansion strategies are best at detecting SNP functional modules in SNP-SNP interaction networks.

SNP tends to interact with its physically close neighbor SNPs and form functional modules. Similar with protein functional module in PPI networks, we define SNP functional module as a group of closely connected SNPs that are together affecting the occurrence and development of disease. MCODE [32] is the earliest protein complex detection method based on seed node expansion strategy. It can easily find out known functional modules in the PPI network. Consequently, by applying MCODE to the SNP-SNP interaction network such modules can be easily found. In this way, computing burden can be greatly reduced while ensuring higher precision. Besides, high-order SNP interactions between or outside SNP functional modules are also considered, however, few of them can be clustered together, due to their low interaction gain values.

### Main effective SNPs detection

In genome-wide single-locus statistic tests, examining each SNP independently for association to the phenotype, statistical tests are different for quantitative traits versus case/control studies. Quantitative traits are generally analyzed by analysis of Variance (ANOVA), which is similar to linear regression. Dichotomous case/control traits are generally analyzed using either contingency table methods or logistic regression [33]. Recently, random forest method [18] which can give variable importance measure is preferred in single-loci association studies. Different to all the methods mentioned above, in this paper, SNP importance is measured by its degree and betweenness centrality. SNPs with higher degree and betweenness centrality are more likely to be associated with the emergence and development of the disease than those with low such values.

### Statistical validation

When selecting potential SNP pairs, only those with higher IG values will be selected. The natural question is how big the probability is when this large score occurs by chance. The probability is estimated by defining a *null hypothesis* which represents, essentially, the scenario that we are not interested in. It is done by shuffling the bases of SNPs on each chromosome. After this shuffling procedure, high-scoring occurrences of the SNP pairs will only appear due to random chance. Then recalculate their IG values. We define our p-value[34] as the probability that a score at least as large as the observed score would occur in data drawn according to the null hypothesis (p-value is set to 0.05 just as researchers usually do.). Unfortunately, as we increase the number of hypotheses in the test, we also increase the likelihood of witnessing a rare event, and therefore, the chance to reject the null hypotheses when it's true (type I error). A *multiple testing correction* procedure is needed to adjust our statistical confidence measures based on the number of tests performed. In this paper, Bonferroni correction is used for multiple testing correction procedure. In short, if you are using a significant threshold of β (in this paper β = 0.05), but you perform *n* separate tests, then the Bonferroni correction deems a score significant only if the corresponding *p*-value is ≤*β* / *n*. The IG values, p-value and corresponding p-values after Bonferroni correction as well as FDR (false discovery rate) on type 2 diabetes (T2D) is presented in Table 4.

**Table 4:** Relationship between α, p-value, FDR using data set of T2D (type 2 diabetes).

α	SNP Interaction Pair Number	p-value	Significant threshold (Bonferroni correction)	FDR
0.001	895654	0.0682	2.37e-9	1.00
0.002	90160	0.0038	2.37e-9	0.89
0.003	34584	1.7e-4	2.37e-9	0.11
0.004	26764	6.87e-6	2.37e-9	0.0054
0.005	24264	2.37e-7	2.37e-9	2.06e-4
0.01	16911	<2.37e-8	2.37e-9	<0.1e-6
0.05	6456	<2.37e-9	2.37e-9	<0.1e-9
0.1	2789	≤2.37e-10	2.37e-9	<0.1e-10

All the SNP interaction pairs in LD are deleted. There are 4595 SNPs in all.4595*4595 = 21114025 pairs of SNPs are tested under NULL hypothesis. The significant threshold after Bonferroni correction = 0.05/21114025 = 2.37e-9.

## Results

### Results on simulated datasets

**Fig 5:**
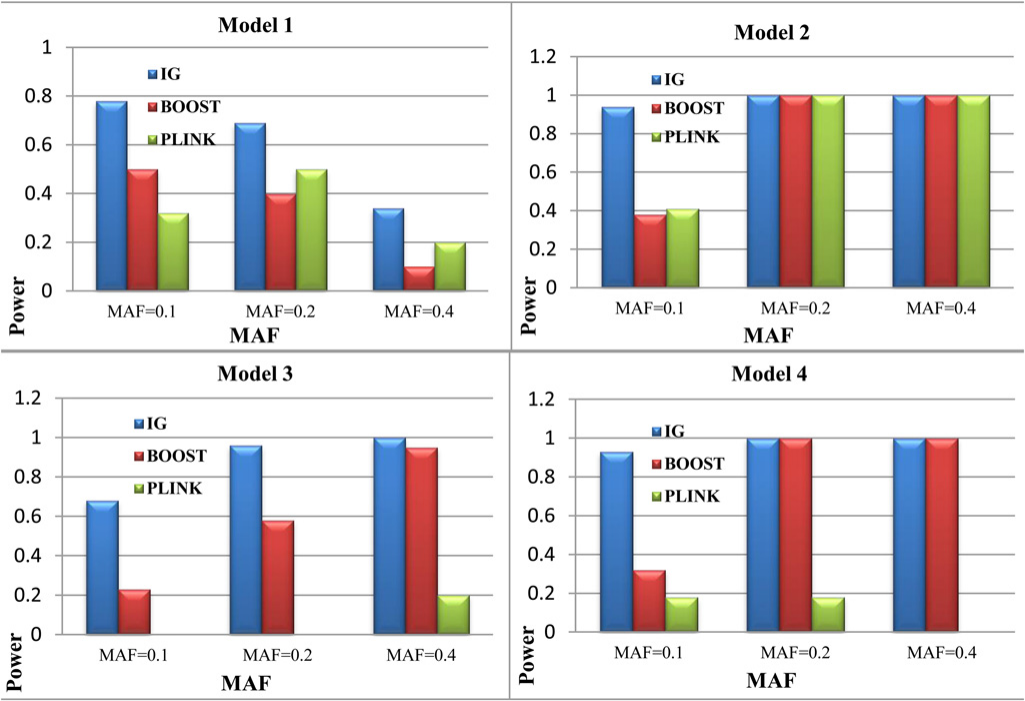
The power comparison between BOOST, PLINK and our method (IG) based on interaction gain. The power is calculated as the proportion of the 100 data sets in which the interactions of the disease-associated SNPs are detected. The absence of bars indicates no power. MAF means minor allele frequency.

The performance of IG (Interaction Gain) is compared with that of BOOST [20] and PLINK [19] with simulated datasets. The power is defined as the ratio of the number of successful identifications and the number of datasets. Results are shown in Fig. 5. Under each parameter setting, 100 datasets are generated, each has 800 cases and 800 controls. We set the interaction gain threshold value *α* = 0.1 (According to our result, interaction gain between two SNPs can be as small as 0, which means two SNPs are independent, and can be as large as 1, which means two SNPs have strong interaction. But the vast majority of interaction gains fell between 0 and 0.1. See Table 4 for more information), parameters of the other two methods use default values. As shown in Fig. 5, within the 300 datasets generated under model 1, IG outperforms the other two methods, and when MAF = 0.1, IG reached the power of 0.78. As for the datasets generated by model 2, all methods performed well when MAF = 0.2 and MAF = 0.4, however, when MAF = 0.1 IG can still maintain higher detection power. As for datasets generated by model 3, PLINK performed poorly when MAF takes values of 0.2 and 0.4. While IG still performs well under each parameter settings. All in all, by using IG almost any form interactions, if they exist, can be captured.

### Results on WTCCC datasets

**Fig 6:**
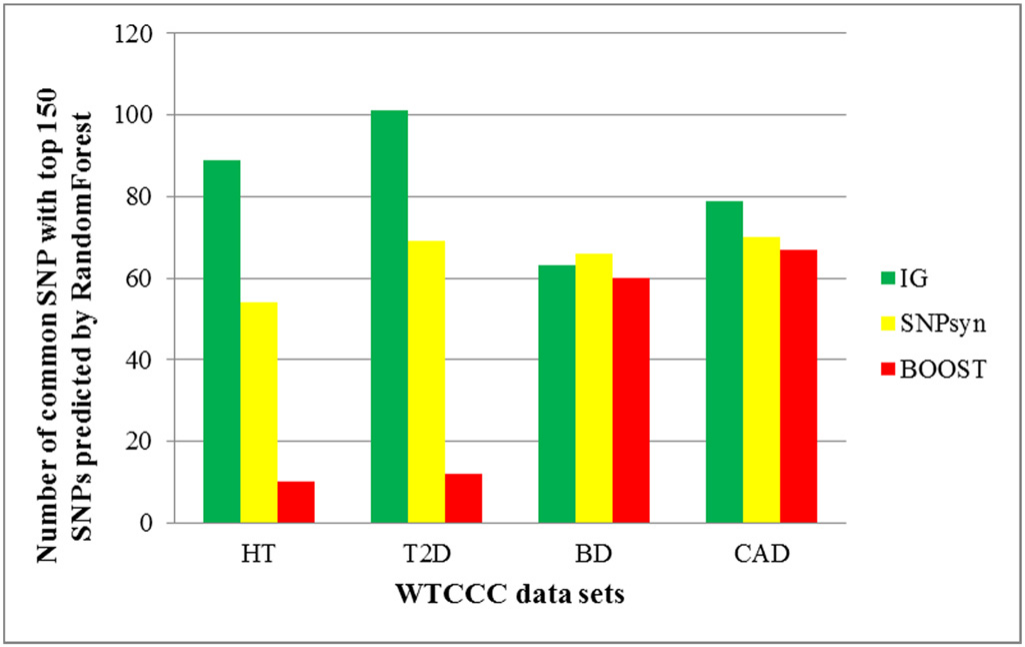
Comparison of the performance of IG, BOOST and SNPsyn in identifying SNPs associated with corresponding disease. During comparison, top 150 SNPs predicted by RandomForest in each WTCCC dataset was used as reference datasets (details are shown in S2 File). The other three methods also took their top 150 most significant SNPs.

With SNPs predicted by Random Forest [18] as benchmark dataset, the performance of IG in identifying main-effect SNPs is compared with that of BOOST and SNPsyn. BOOST [20] creates multiple paths in which the visited SNP functional modules tend to be statistical associated with disease, then harvests those significant SNP functional modules which pass the statistical tests. It greatly reduces the number of SNPs tested. SNPsyn detects synergy SNP pairs taking use of mutual information, and can also give SNP importance measurement. The performance of Random Forest in genetic data analysis has been investigated which has been shown to out-perform Fisher’s exact test [35] and many other methods [18] as a screening tool when interactions are present. For this reason and lack of other high confidence reference datasets, top ranked 150 SNPS predicted by Random Forest in each dataset were used as benchmark datasets (Shown in S2 File). We measure the importance of each SNP by measuring its degree and betweenness centrality. Threshold values of all the methods were adjusted in order to filter out almost the same number of disease associated SNPs. Results were shown in Fig. 6.

To grow RFs (Random Forests) and estimate SNP importance value, the randomForest package by Liaw and Wiener is used. 500 trees were grown with a random selection of 20 variables per node. On average, each variable was contained in a RF 84 times. The average importance scores across all RFs were used as the global importance value of a variable. In each result only top 150 most important SNPs were selected. As shown in Fig. 6, IG can match more SNPs predicted by Random Forest than other two methods. However, even in T2D, of the 150 SNPs predicted by IG only 100 of them are also predicted by Random Forest. It does not mean that Random Forest performs better than IG. For example, of the SNPs predicted by IG but not of Random Forest, SNP rs2877832 (PubMed: 17903298) was proved to be associated with type 2 diabetes; SNPs rs688034 (PubMed: 1754300), rs2269511 (PubMed: 18794856) were shown to be associated with hypertension, all of them cannot be identified by Random Forest. By using degree and betweenness centrality as metric to select disease associated SNPs, IG achieved much higher accuracy than BOOST and SNPsyn. As shown in Fig. 6, compared with BOOST, SNPsyn can find out more disease related mutations. Because by taking advantage of mutual information, SNPs associated with disease are more likely to be selected.

**Fig 7:**
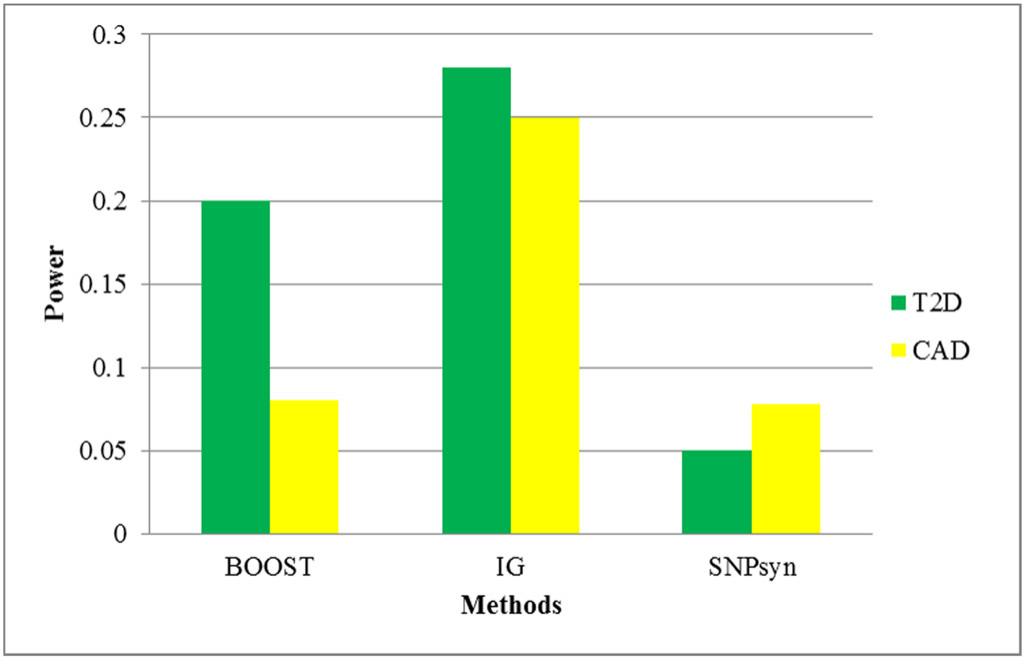
The power comparison of BOOST, IG, SNPsyn in detecting disease associated SNP-SNP interaction pairs using data sets of T2D and CAD. The power is defined as the ratio of disease related genes and all mapped genes.

The power of IG, BOOST and SNPsyn in detecting disease related low-order (pairwise) SNP interactions was compared using the T2D and CAD datasets. The power is defined as the ratio of disease related genes and all mapped genes. For simplicity, only top ranked 100 SNP-SNP interaction pairs are selected and compared in each dataset. To compute the power, SNPs contained in each of the 100 pairs of SNP-SNP interactions are mapped on genes using SNP4disease (SNP4Disease (http://snp4disease.mpi-bn.mpg.de/index.php) is a knowledge database which is constructed using data and literature mining techniques to incorporate data from various publicly-available databases. It provides the information about disease associated SNPs (genes) and their potential biological impacts.). Mapping results are shown in supplementary material (S1 File). For example, 121 SNPs contained in the top ranked 100 SNP-SNP interaction pairs predicted by IG. Those SNPs are mapped on 10 genes. Three (SLC7A7, C14orf—, KCNH5) of them were validated have relations with T2D. So the power of IG considering this data set is 3/10. Comparison results are shown in Fig. 7. More information can be got from supplementary material (S1 File). As can be seen from Fig. 7, IG achieved much higher power than the other two methods with T2D and CAD datasets.

As for high-order SNP interaction detection, MCODE was applied to the SNP-SNP interaction networks constructed from the WTCCC HT and T2D datasets. Since there is no reference high-order SNP interaction datasets, the performance of MCODE in detecting SNP functional modules is evaluated by counting the number of SNPs within each module which have been researched by others. The result was presented in Table 5. Some SNP functional modules detected from the network constructed from IG detection results are shown in S6 Fig., S7 Fig., S8 Fig., S9 Fig., S10 Fig. As shown in Table 5, SNPs within the same SNP functional module tend to come from the same gene or from genes which are close to each other. Single mutation within the gene may have few bad effects on it, for a disease to occur relevant genes may need to experience substantial changes. That is the reason why SNPs tend to form SNP functional modules (high-order SNP interactions) and together contribute to disease development. Apart from the SNP interactions exist in the same SNP functional modules, higher order interactions occur between SNP functional modules or outside SNP functional modules were also considered. Unfortunately, due to the low interaction value between those SNPs, few high-order SNP interactions can be detected. Considering this, finding high-order SNP interactions by finding SNP functional modules can ensure the accuracy and stability of the final results.

**Table 5:** Five SNP functional modules detected by MCODE using HT.

SNP functional module ID	SNP members	Genes Involved	SNPs In PubMed	Functional module
1	rs2017874,rs738536,rs738387,rs8138930,rs4820483,rs738378,rs13433641,rs4822208,rs2273142	PACSIN2, ARFGAP3	rs738536(ID: 20018033)	See S1 Fig.
2	rs9605422,rs2165971,rs2075453,rs4819644,rs2075444,rs1057721,rs2016042,rs11917,rs2075455,rs873387	MICAL3	no	See S2 Fig.
3	rs7289941,rs1034589,rs2413035,rs5753659,rs2106294,rs5998067,rs6518752,rs9609297,rs7287267	RNF185, LIMK2,SFI1,EIF4ENIF1	rs2106294(ID: 21150874)	See S3 Fig.
4	rs1569492,rs5757187,rs1946990,rs6519120,rs5757203,rs1056610,rs10135,rs5757133,rs138702,rs6001173,rs4820335,rs138703	JOSD1, SUN2, TOMM22, LOC646851, DMC1	rs5757133(ID: 20084279)	See S4 Fig.
5	rs5770112,rs916251,rs2688171,rs2253004,rs2688155,rs5770111,rs2688148,rs5769491	NULL	rs5770111(ID: 17357082)	See S5 Fig.

SNP members are the SNPs that falls into the same SNP complex. SNPs in the PubMed represent the SNPs in the SNP functional module that have been researched by other people.

## Discussion and Conclusion

### Parameters discussion

**Fig 8:**
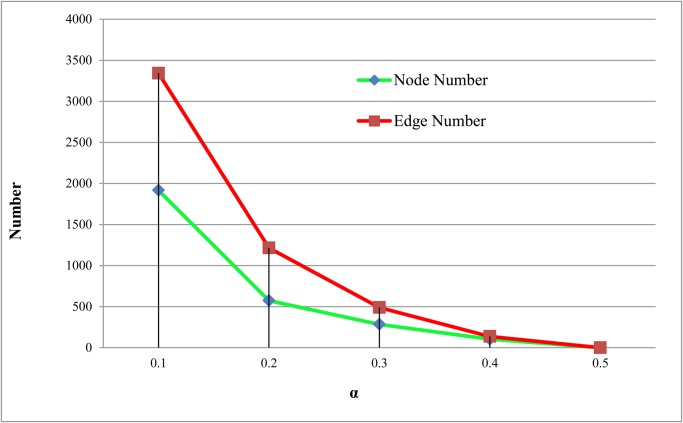
Relationship between *α* and node and edge number of the SNP interaction network constructed. *α* is the threshold value.

In the previously sections, threshold value *α* is set to 0.1. In this section, the effect of *α* on network size is studied (the number of SNP pairs). Results are shown in Fig. 8. According to our calculations, interaction gain between two SNPs can be as small as 0, which means two SNPs are independent. In fact, the vast majority of interaction gain values fall within the range between [0,0.1]. The relationship between α, p-value and FDR is presented in Table 4 (using dataset of T2D). According to Table 4, when α = 0.1 we can get more reliable interactions with lower p-value and FDR. So we choose *α* = 0.1 as our threshold value, because SNPs with higher interaction gain values are more likely to have true interactions.

### Conclusion

Given numerous GWA studies that have recently or are currently being performed, it is clear that, genome wide interaction research will be the natural next step following the single-locus testing. In fact, there are growing interests in developing and applying computational and statistical approaches for SNP interactions detection. Detecting low-order and high-order SNP interactions is considered important for deeply understanding of relationships between mutations and diseases. In this paper, both low-order and high-order SNP interactions were detected with the proposed methods. Besides, we proposed to measure main-effect SNPs from a new point of view. More specifically, a pairwise (or low-order) interaction detection method IG (Interaction Gain) working in parallel computing was designed by taking advantage of entropy and information gain in information theory. High-order SNP interactions were proposed to be detected by finding functional modules of the network constructed from IG detection results. Main-effect SNPs were measured by their degree and betweenness centrality. Performance comparisons were done between IG, PLINK, BOOST and SNPsyn on both simulated datasets and WTCCC datasets. With proposed methods more reliable interactions were discovered with higher accuracy and better performance. Knowledge of major SNP interactions provides insight into the relationship of complex pathways and also highlights key genes that could be targets for therapy or drug targets. The research will advance the complex diseases research by providing more reliable SNP interactions. While we have provided internal validation for a subset of the tested SNP interactions in this study, corresponding interactions require further cross validations in other populations.

## Supporting Information

S1 Fig One of the SNP functional modules detected by MCODE within the network constructed from IG detection results using HT (hypertension) dataset. The nodes represent SNPs, and the edge between any two SNPs indicates there exist interaction between them. SNPs within the same functional module tend to together affecting the emergence and development of disease. All of the SNPs and edges formed the high-order SNP interactions we are interested in.
(TIF)

S2 Fig One of the SNP functional modules detected by MCODE within the network constructed from IG detection results using HT (hypertension) dataset. This figure has the same meaning with S1 Fig.
(TIF)

S3 Fig One of the SNP functional modules detected by MCODE within the network constructed from IG detection results using HT (hypertension) dataset. This figure has the same meaning with S1 Fig.
(TIF)

S4 Fig One of the SNP functional modules detected by MCODE within the network constructed from IG detection results using HT (hypertension) dataset. This figure has the same meaning with S1 Fig.
(TIF)

S5 Fig One of the SNP functional modules detected by MCODE within the network constructed from IG detection results using HT (hypertension) dataset. This figure has the same meaning with S1 Fig.
(TIF)

S6 Fig One of the SNP functional modules detected by MCODE within the network constructed from IG detection results using T2D (type 2 diabetes) dataset. This figure has the same meaning with S1 Fig.
(TIF)

S7 Fig One of the SNP functional modules detected by MCODE within the network constructed from IG detection results using T2D (type 2 diabetes) dataset. This figure has the same meaning with S1 Fig.
(TIF)

S8 Fig One of the SNP functional modules detected by MCODE within the network constructed from IG detection results using T2D (type 2 diabetes) dataset. This figure has the same meaning with S1 Fig.
(TIF)

S9 Fig One of the SNP functional modules detected by MCODE within the network constructed from IG detection results using T2D (type 2 diabetes) dataset. This figure has the same meaning with S1 Fig.
(TIF)

S10 Fig One of the SNP functional modules detected by MCODE within the network constructed from IG detection results using T2D (type 2 diabetes) dataset. This figure has the same meaning with S1 Fig.
(TIF)

S1 File WTCCC-SNP-SNP-comparison result. This file shows the mapping results of BOOST IG SNPsyn as well as the prediction power of the three methods.
(XLSX)

S2 File WTCCC-Results-Single-SNP-RandomForest-top150. This file shows the details of the top 150 SNPs predicted by RandomForest in each WTCCC dataset.
(XLSX)
