# Supplementary figures and images for: Research on Single Nucleotide Polymorphisms Interaction Detection from Network Perspective
Source: PLoS One. 2015 Mar 12;10(3):e0119146. doi: 10.1371/journal.pone.0119146 (PMC4357495; doi:10.1371/journal.pone.0119146)

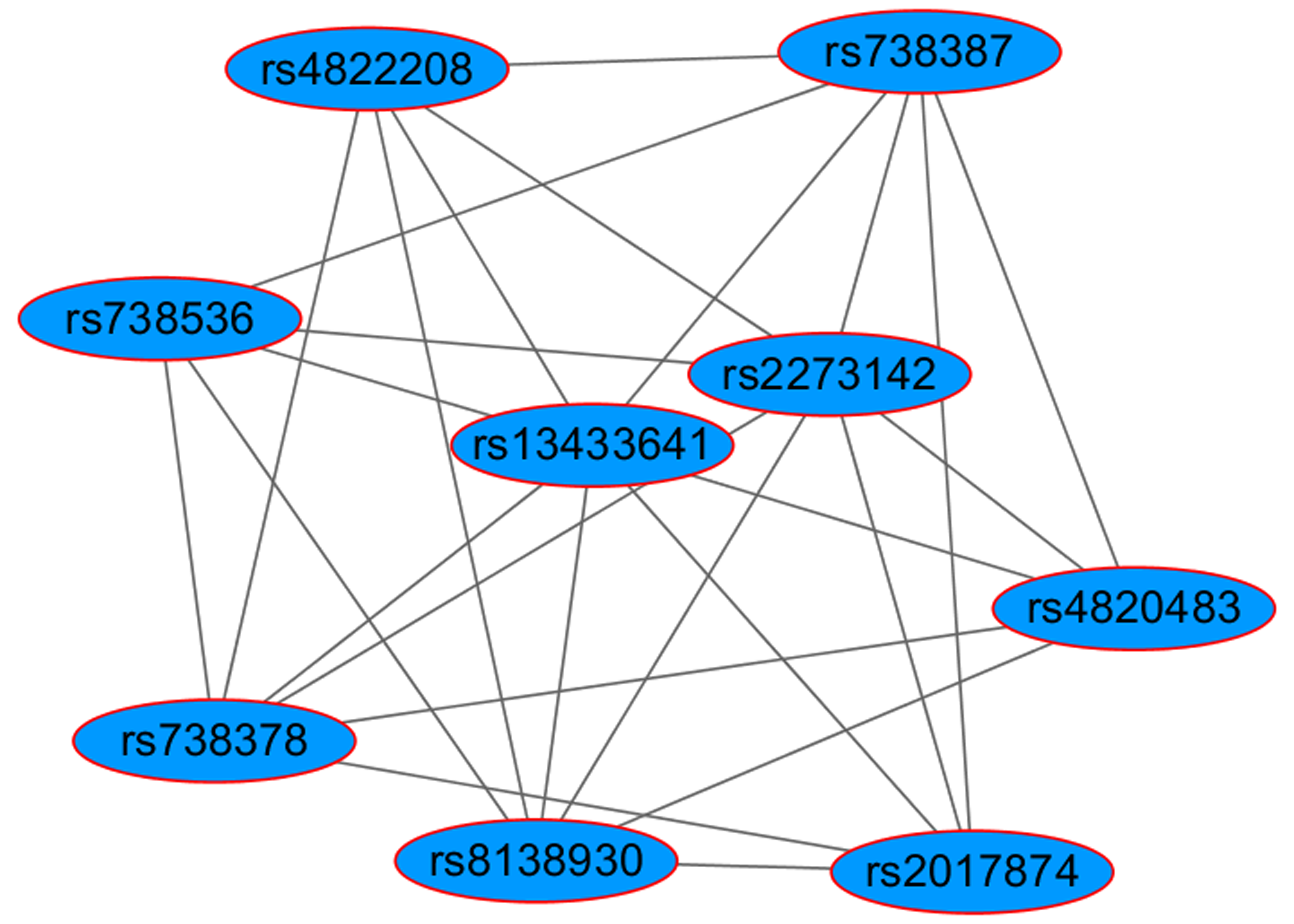

Supplement: S1 Fig — The nodes represent SNPs, and the edge between any two SNPs indicates there exist interaction between them. SNPs within the same functional module tend to together affecting the emergence and development of disease. All of the SNPs and edges formed the high-order SNP interactions we are interested in. (TIF) [file pone.0119146.s001.tif]

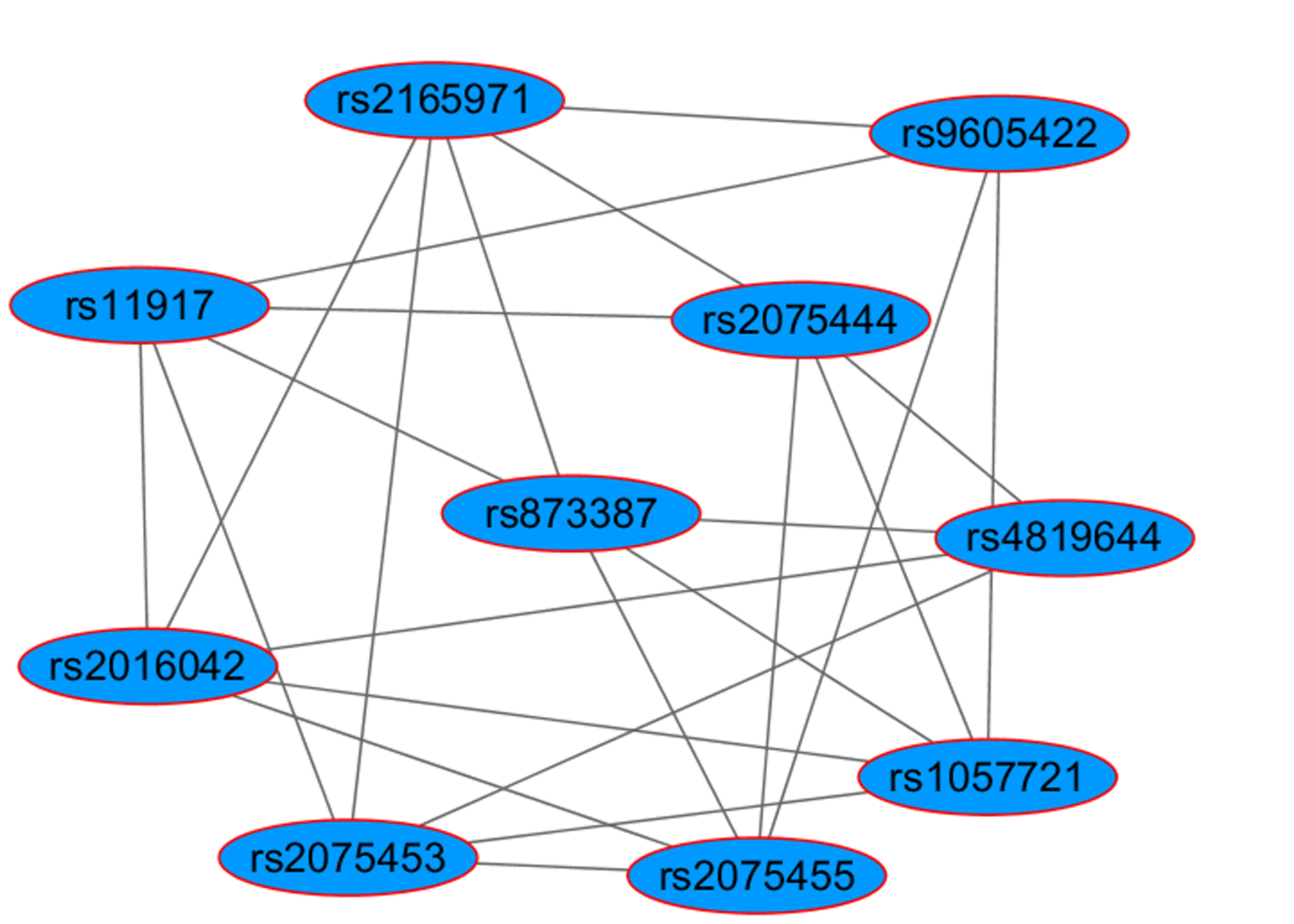

Supplement: S2 Fig — This figure has the same meaning with S1 Fig. (TIF) [file pone.0119146.s002.tif]

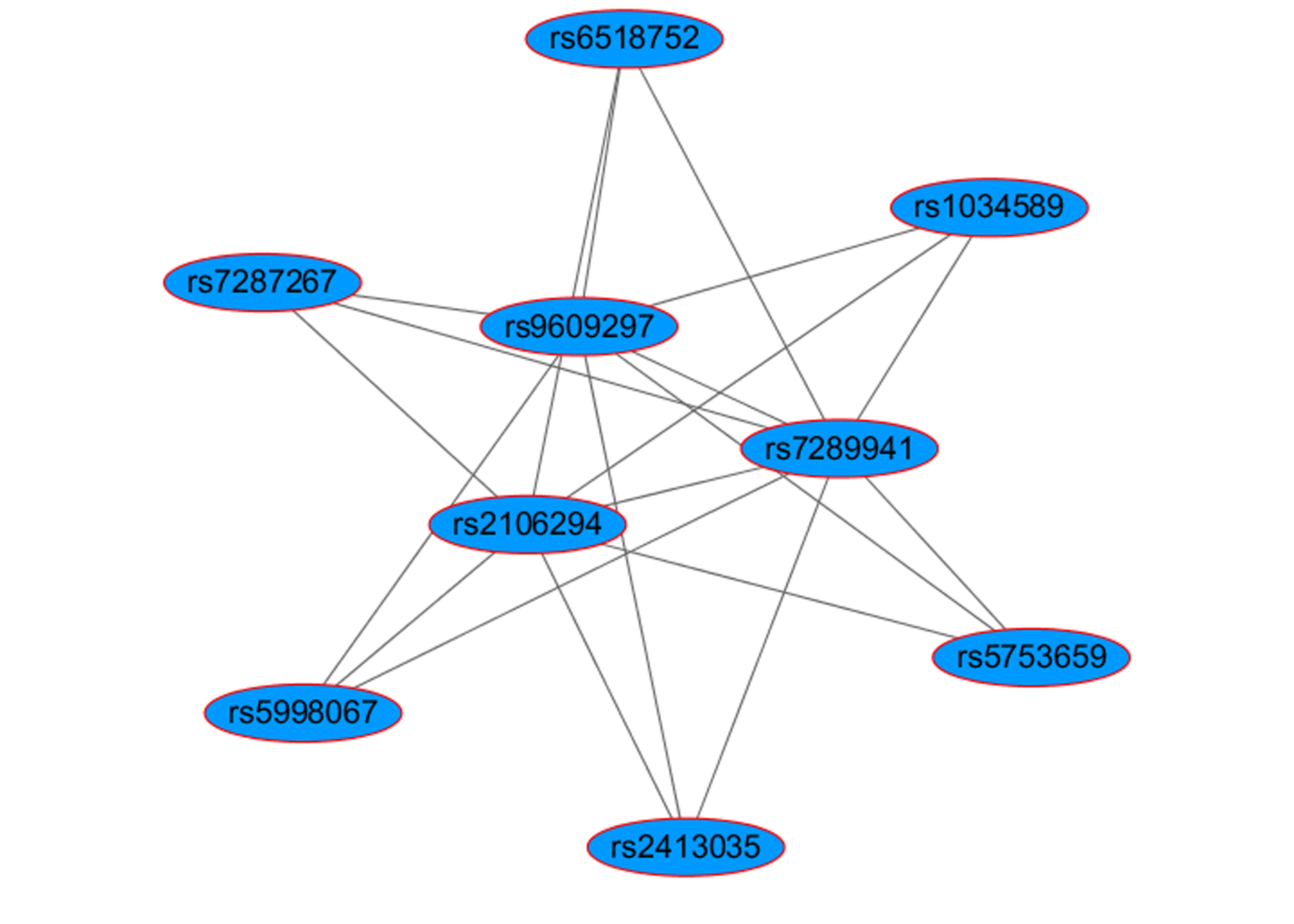

Supplement: S3 Fig — This figure has the same meaning with S1 Fig. (TIF) [file pone.0119146.s003.tif]

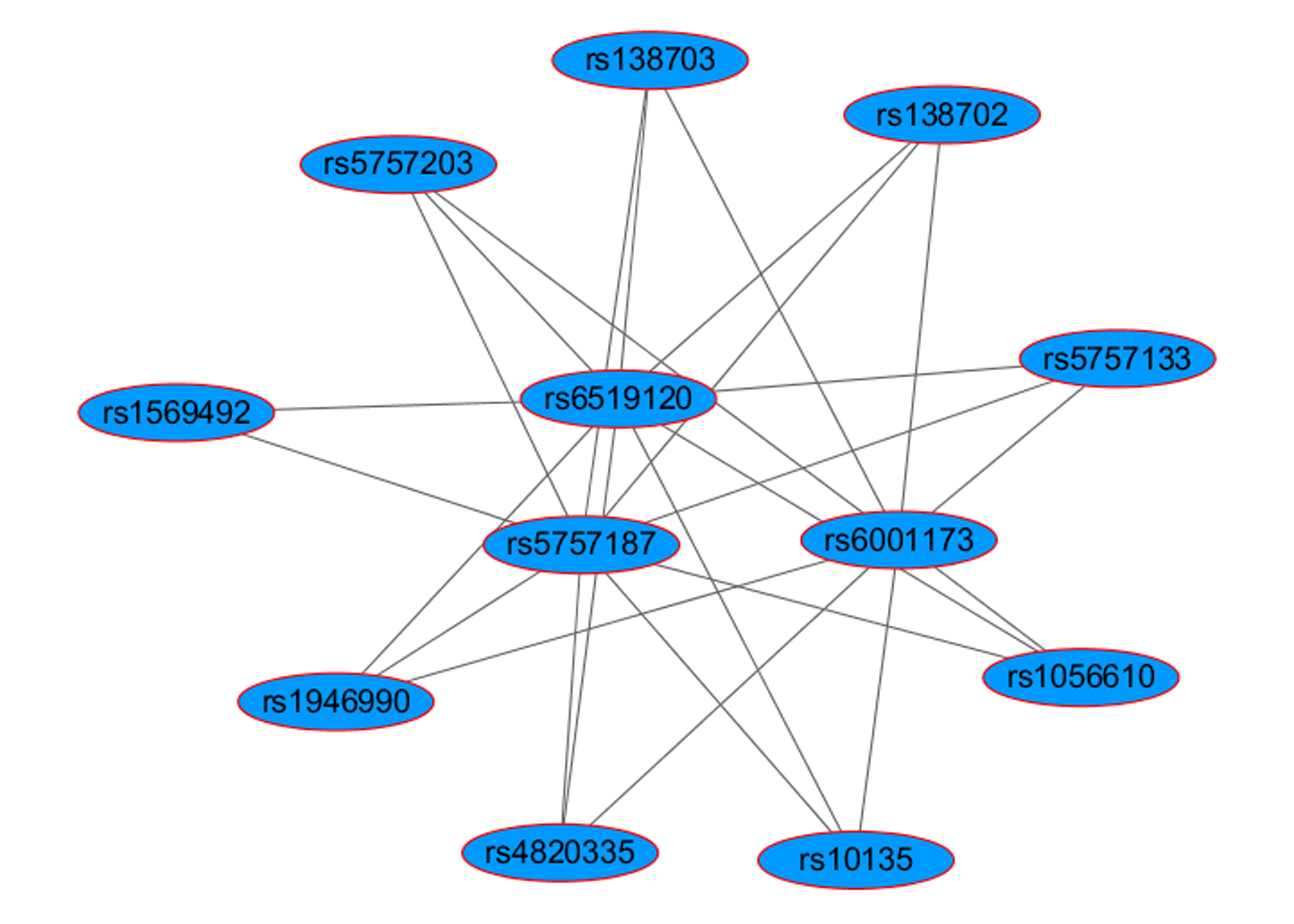

Supplement: S4 Fig — This figure has the same meaning with S1 Fig. (TIF) [file pone.0119146.s004.tif]

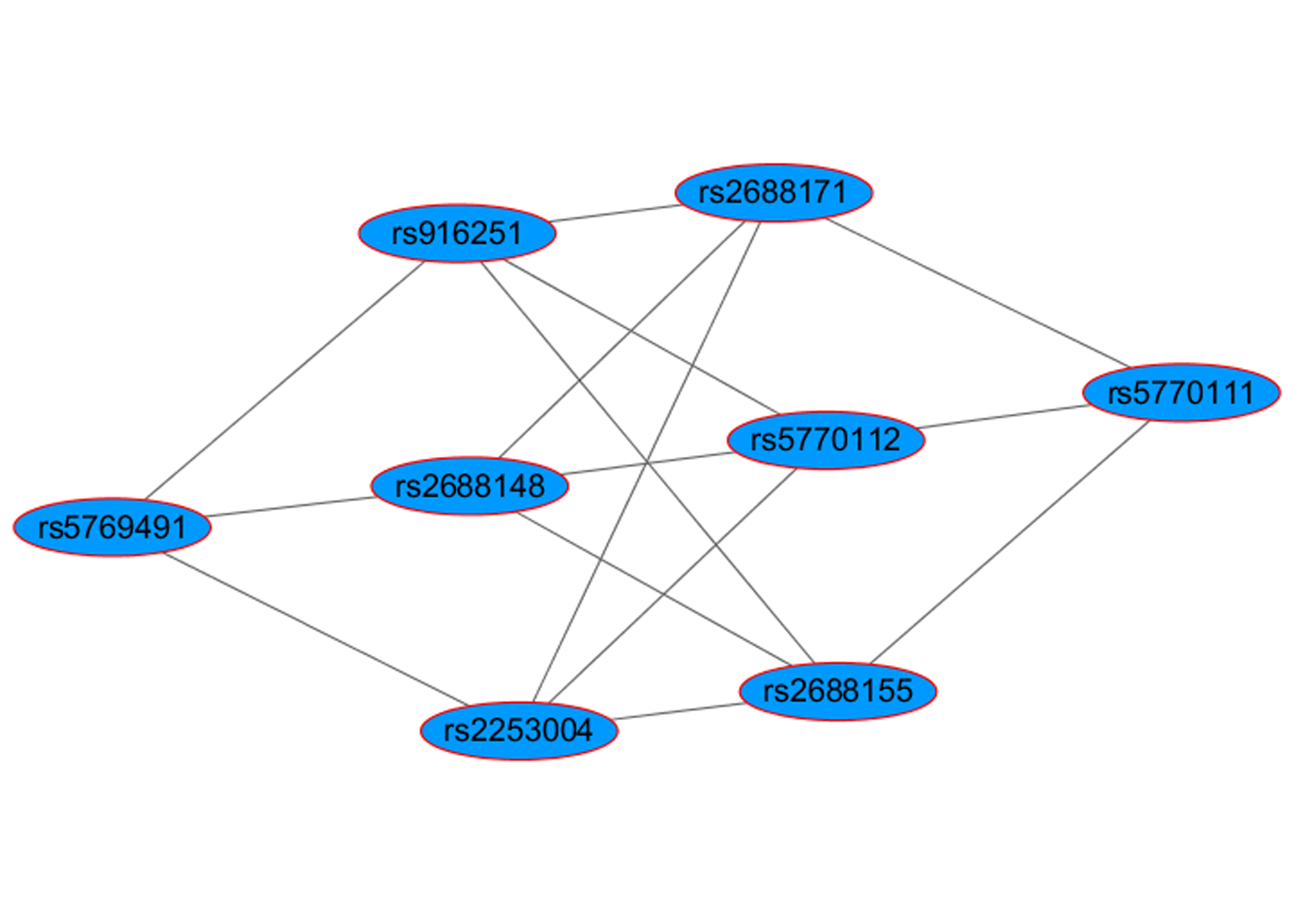

Supplement: S5 Fig — This figure has the same meaning with S1 Fig. (TIF) [file pone.0119146.s005.tif]

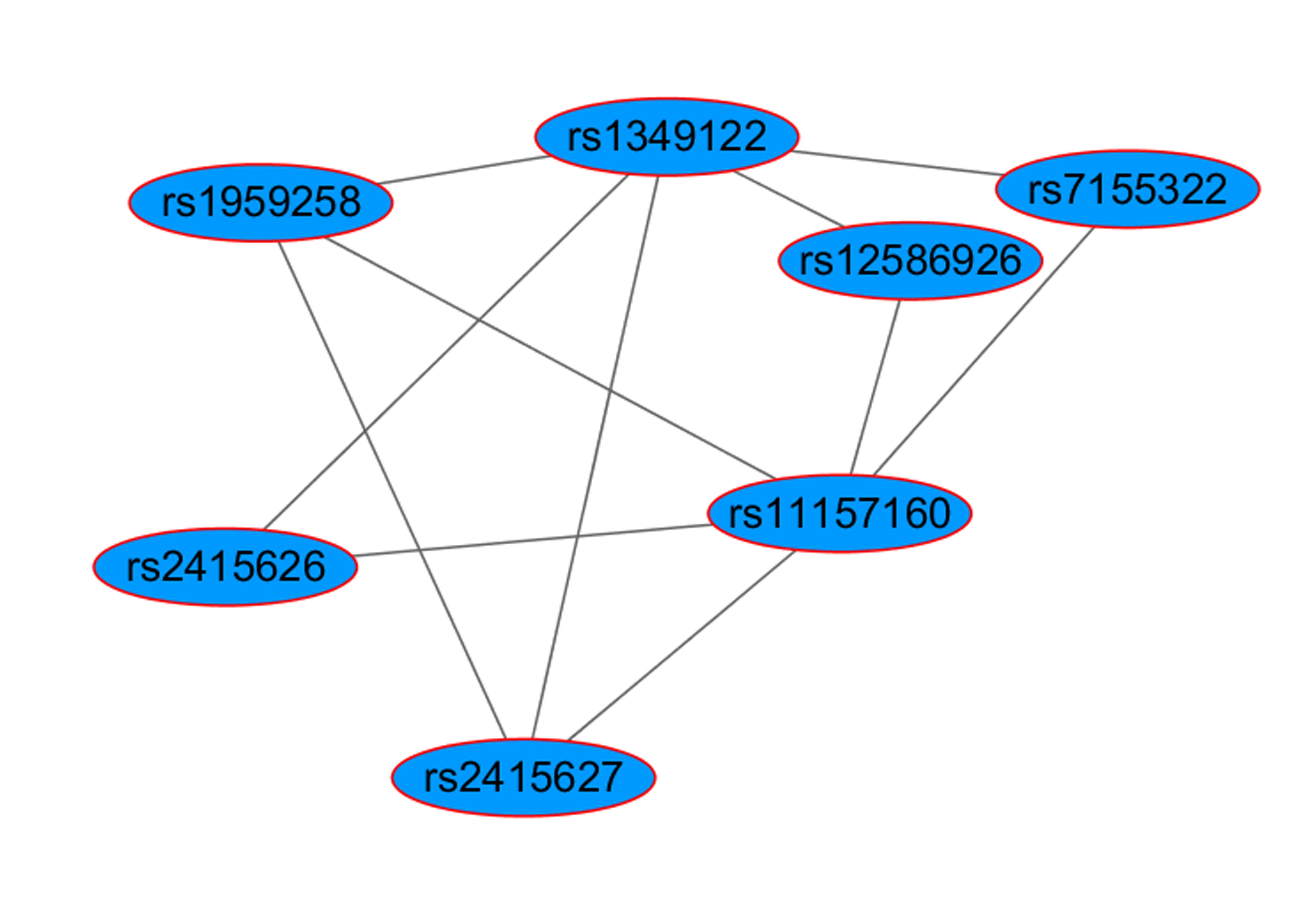

Supplement: S6 Fig — This figure has the same meaning with S1 Fig. (TIF) [file pone.0119146.s006.tif]

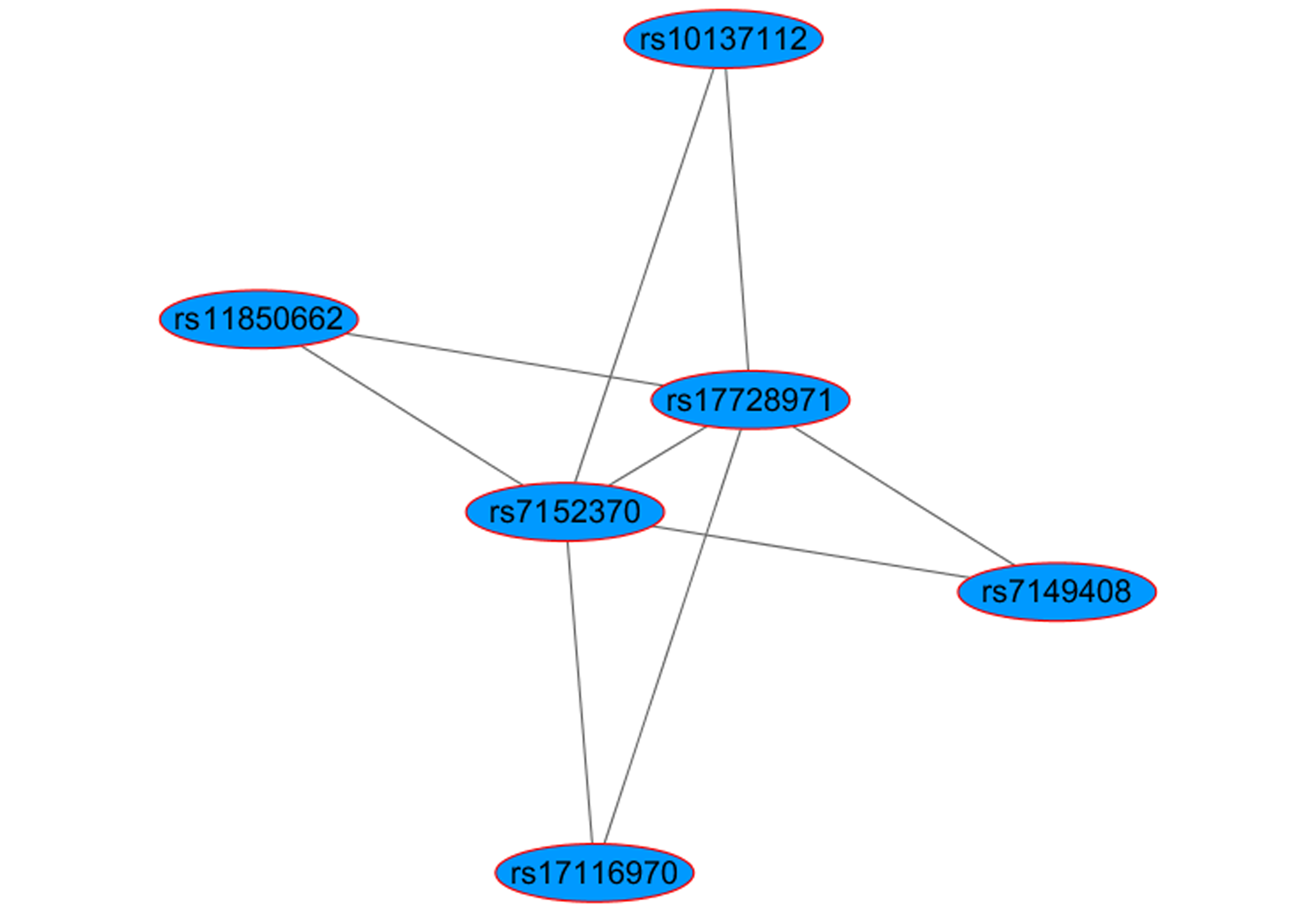

Supplement: S7 Fig — This figure has the same meaning with S1 Fig. (TIF) [file pone.0119146.s007.tif]

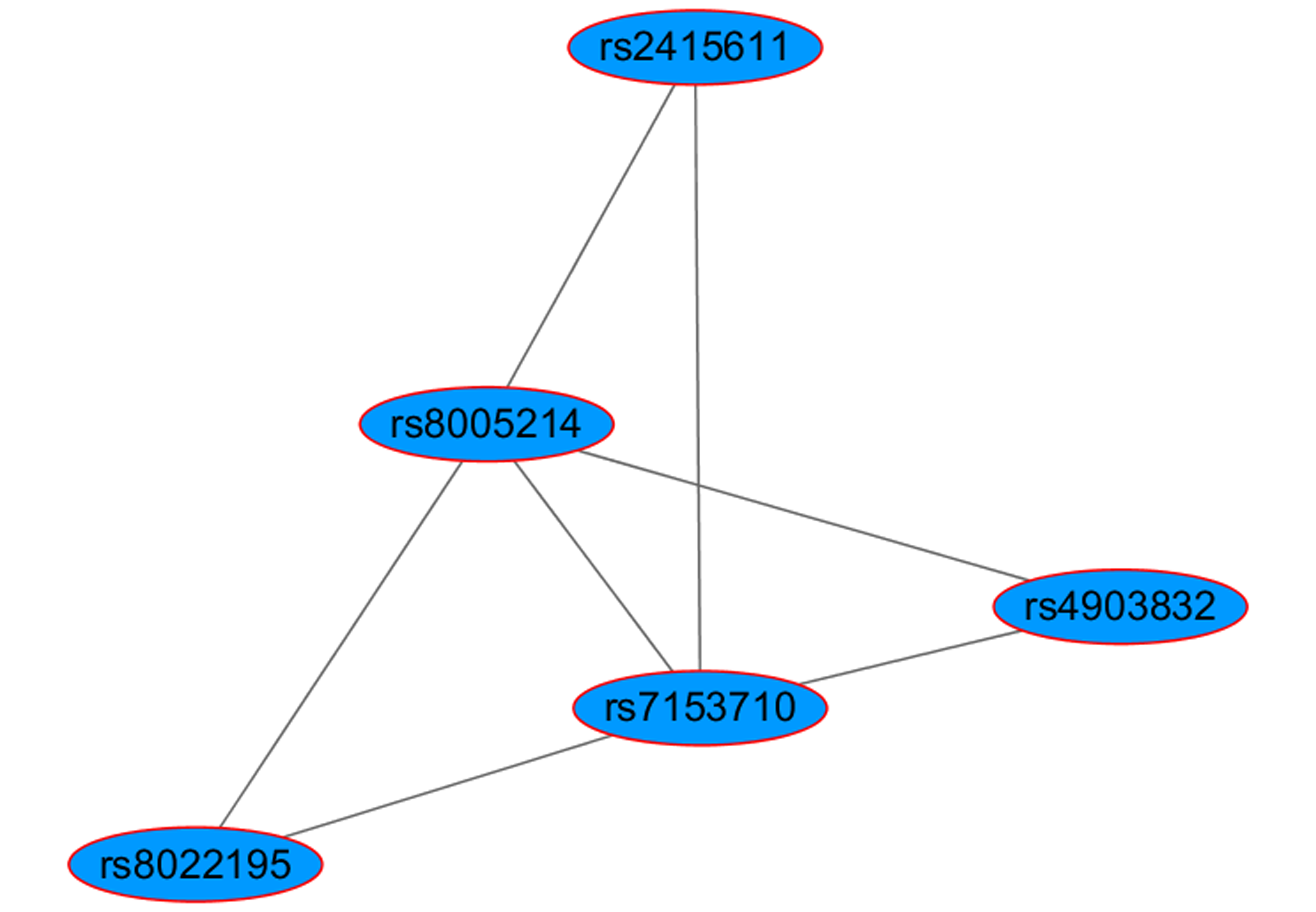

Supplement: S8 Fig — This figure has the same meaning with S1 Fig. (TIF) [file pone.0119146.s008.tif]

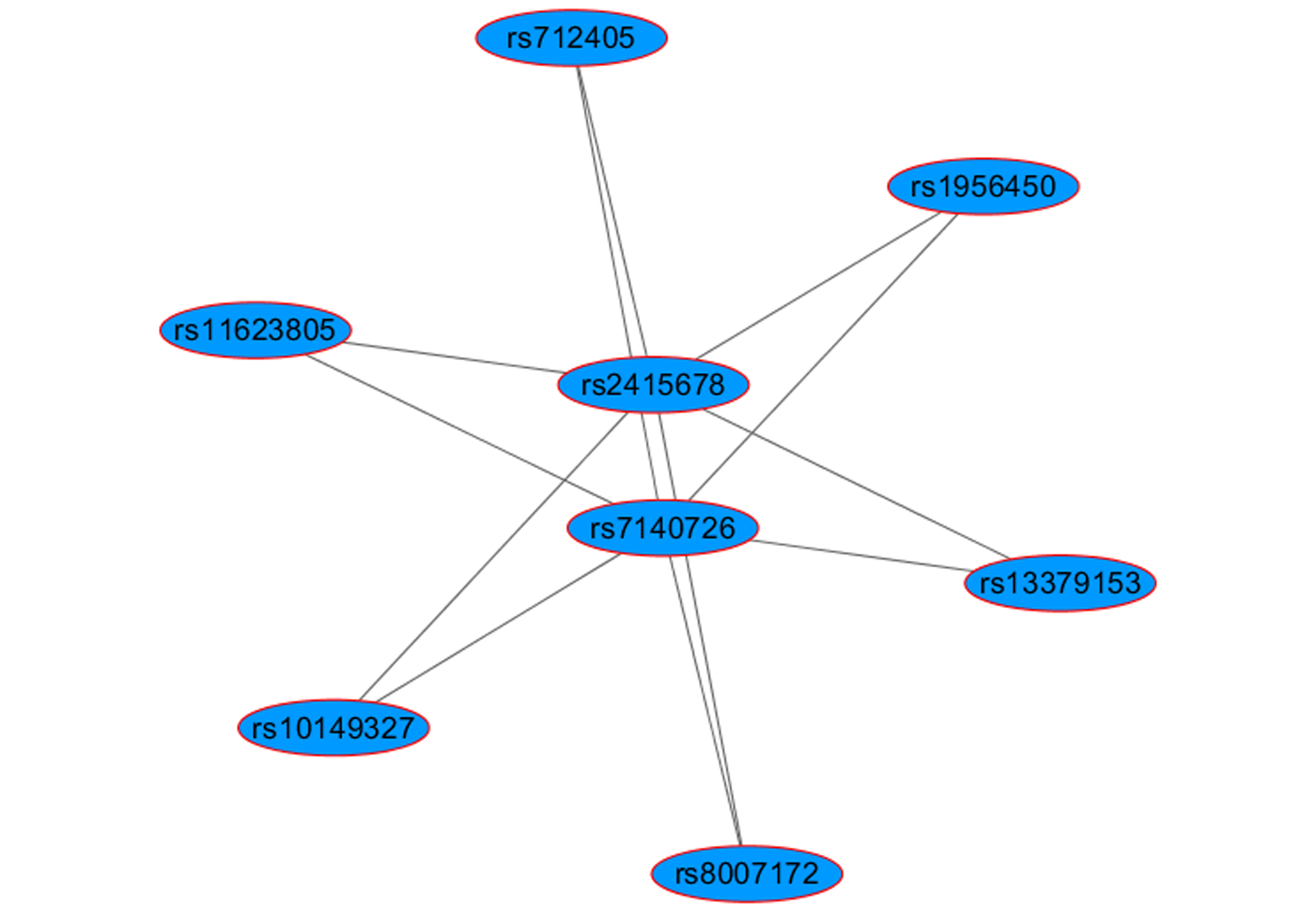

Supplement: S9 Fig — This figure has the same meaning with S1 Fig. (TIF) [file pone.0119146.s009.tif]

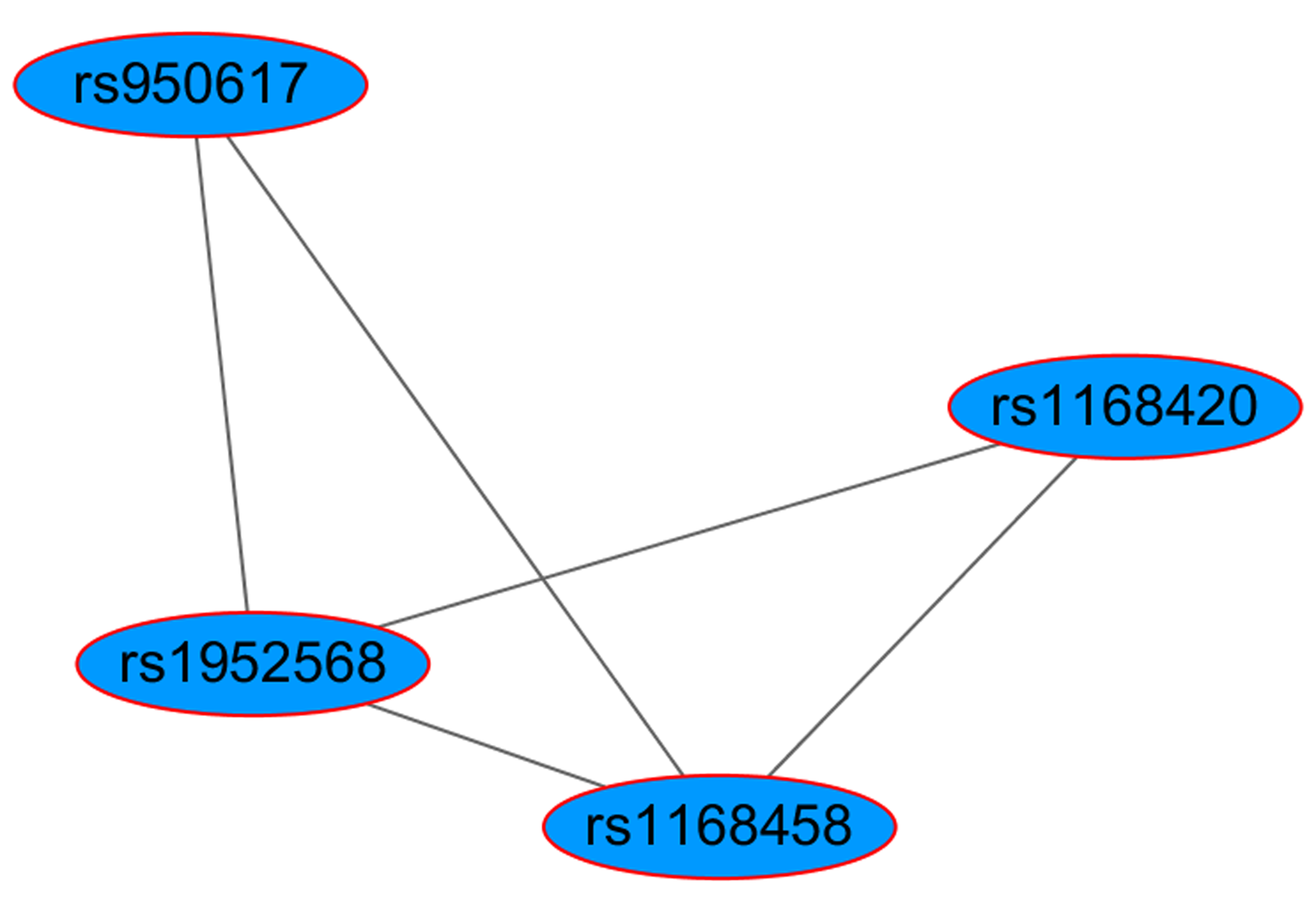

Supplement: S10 Fig — This figure has the same meaning with S1 Fig. (TIF) [file pone.0119146.s010.tif]
